# Oral immunization with an attenuated *Salmonella* Gallinarum mutant as a fowl typhoid vaccine with a live adjuvant strain secreting the B subunit of *Escherichia coli* heat-labile enterotoxin

**DOI:** 10.1186/1746-6148-9-96

**Published:** 2013-05-06

**Authors:** Byung Woo Jeon, Rahul M Nandre, John Hwa Lee

**Affiliations:** 1 College of Veterinary Medicine, Chonbuk National University, Jeonju 561-756, Republic of Korea

## Abstract

**Background:**

The *Salmonella* Gallinarum (SG) *lon/cpxR* deletion mutant JOL916 was developed as a live vaccine candidate for fowl typhoid (FT), and a SG mutant secreting an *Escherichia coli* heat-labile enterotoxin B subunit (LTB), designated JOL1229, was recently constructed as an adjuvant strain for oral vaccination against FT. In this study, we evaluated the immunogenicity and protective properties of the SG mutant JOL916 and the LTB adjuvant strain JOL1229 in order to establish a prime and boost immunization strategy for each strain. In addition, we compared the increase in body weight, the immunogenicity, the egg production rates, and the bacteriological egg contamination of these strains with those of SG 9R, a widely used commercial vaccine.

**Results:**

Plasma IgG, intestinal secretory IgA (sIgA), and cell-mediated responses were significantly induced after a boost inoculation with a mixture of JOL916 and JOL1229, and significant reductions in the mortality of chickens challenged with a wild-type SG strain were observed in the immunized groups. There were no significant differences in increases in body weight, cell-mediated immune responses, or systemic IgG responses between our vaccine mixture and the SG 9R vaccine groups. However, there was a significant elevation in intestinal sIgA in chickens immunized with our mixture at 3 weeks post-prime-immunization and at 3 weeks post-boost-immunization, while sIgA levels in SG 9R-immunized chickens were not significantly elevated compared to the control. In addition, the SG strain was not detected in the eggs of chickens immunized with our mixture.

**Conclusion:**

Our results suggest that immunization with the LTB-adjuvant strain JOL1229 can significantly increase the immune response, and provide efficient protection against FT with no side effects on body weight, egg production, or egg contamination.

## Background

Fowl typhoid (FT) is a severe septicemic bacterial disease in poultry caused by *Salmonella enterica* serovar Gallinarum biovar Gallinarum (SG), which can be horizontally or vertically transmitted [1,2]. Due to a variety of factors, both the morbidity and mortality of FT are highly variable, and a recent study reported mortality rates in excess of 60% in experimentally infected chickens [3]. Although strong eradication programs have largely or completely controlled the disease in North America and several European countries, SG is still of considerable economic importance in many countries throughout South America, Africa, and Asia [2]. Attenuated rough strains of SG strain 9 (SG 9R) have been used as live vaccines for chickens since the 1950s [4]. SG 9R strain has been still accepted as the most effective live vaccine against FT infection in endemic countries [5,6]. Unfortunately, invasive administration of the 9R vaccine using syringes is both uneconomical and labor intensive.

Recently, an SG mutant, JOL916 was constructed as a live vaccine candidate for FT by deleting the *lon* and *cpxR* genes, which are related to the host-pathogen interaction [7]. An attenuated SG strain, JOL1229, which secretes *Escherichia coli* heat-labile enterotoxin B subunit (LTB), was also constructed as an adjuvant strain for oral vaccination against FT [8]. A non-toxic LTB binds to the monosialotetrahexosylganglioside (GM1) ganglioside receptors of target cells, and induces the cell to take up the whole toxin, which allows for the utilization of LTB as an adjuvant for foreign antigens [9]. LTB acts on modulating cell-mediated immune responses (Th1) and humoral immune responses (Th2) [10]. Oral immunization with a mixture of four parts JOL916 and one part JOL1229 was reported to enhance the immune response and provide efficient protection against FT in 6-week-old chickens [8]. In order to maintain immunogenicity till the egg laying period in inoculated chickens, it will be necessary to optimize this novel oral immunization strategy through a prime-boost strategy using the LTB adjuvant strain. In the present study, we aimed to establish prime and boost immunization strategies using the SG mutant JOL916 and the LTB adjuvant strain JOL1229, and we examined both the immune responses and protection efficacy. In addition, changes in body weight, immunogenicity, egg production rates, and bacteriological contamination after immunization with the JOL916-JOL1229 mixture were compared with a SG 9R-immunized group.

## Methods

### Experimental animals

Female Hy-Line Brown layer chickens were used in this study. Chickens were provided with ad libitum access to water and antibiotic-free feed. Animal experiments were conducted with the approval of the Chonbuk National University Animal Ethics Committee in accordance with the guidelines of the Korean Council on Animal Care (CBU 2011–0017).

### Bacterial strains

JOL916, an SG mutant with the *lon* and *cpxR* genes deleted, was used as the live vaccine strain [7], and JOL1229, which carries a recombinant Asd^+^ plasmid harboring the *eltB* gene expressing LTB protein, was used as the live adjuvant strain [8]. For comparative study with a commercial SG vaccine, the Nobilis® SG9R vaccine (Intervet International, Boxmeer, The Netherlands) was used. The wild-type SG strain JOL420, provided by the Animal, Plant, and Fisheries Quarantine and Inspection Agency of Korea, was used for the challenge inoculation to induce acute systemic FT.

### Experimental design

Experiment 1: For the optimization of the prime-boost immunization strategy using JOL1229, thirty female chickens were divided into three groups (*n* = 10 per group). Chickens were orally primed at 6 weeks of age and orally boosted at 16 weeks of age. The immunization protocols are described in Table 1. Each bird in the immunized groups was orally inoculated with 200 μl of bacterial suspension containing 1 × 10^8^ colony-forming units (CFU), and each chicken in the control group was given 200 μl of phosphate-buffered saline (PBS). Group A chickens were primed and boosted with a mixture consisting of four parts JOL916 (0.8 × 10^8^ CFU) and one part JOL1229 (0.2 × 10^8^ CFU), group B chickens were primed with a mixture consisting of four parts JOL916 (0.8 × 10^8^ CFU) and one part JOL1229 (0.2 × 10^8^ CFU), and were boosted with JOL916, and group C chickens were inoculated with PBS as the control group. At four weeks after boost-immunization, hens were orally challenged with 1 × 10^6^ CFU of the wild-type SG strain, JOL420.

**Table 1:** Immunization, mortality, and gross lesions in the internal organs of chickens after challenge in experiment 1

**Group**	**Immunization**^**a**^		***n***	**Mortality (%)**	**Gross lesion**^**b**^			
					**Liver**		**Spleen**	
	**Prime**	**Booster**			**EN**^**c**^	**NF**^**d**^	**EN**	**NF**
A	JOL916/JOL1229	JOL916/JOL1229	10	10^*^	0.7±1.1^*^	0.4±1.0^*^	0.4±1.0^*^	0.7±0.9^*^
B	JOL916/JOL1229	JOL916	10	10^*^	0.6±1.0^*^	0.8±1.1^*^	0.7±1.2^*^	0.5±1.0^**^
C	PBS	PBS	10	70	2.1±1.4	2.1±1.4	2.1±1.4	2.3±1.3

^a^ JOL916 is a vaccine strain and JOL1229 is an LTB protein-secreting strain for adjuvant. JOL916/JOL1229 is a mixture consisting of 4 parts JOL916 and 1 part JOL1229. The dose of immunization was 1 × 10^8^ CFU in a volume of 200 μl.

^b^ Gross lesion score (mean ± SEM).

^c^ Enlargement.

^d^ Necrotic foci.

Asterisks indicate a significant difference compared to the control group (^*^p < 0.05, ^**^p < 0.01). *n*, numbers of the chickens used.

Experiment 2: For the comparison of the safety and efficacy of our SG vaccine candidate with the commercial vaccine SG 9R, thirty-six chickens were divided into three groups. Group I chickens were orally primed and boosted with 200 μl of bacterial suspension containing 1 × 10^8^ CFU of a mixture consisting of four parts JOL916 (0.8 × 10^8^ CFU) and one part JOL1229 (0.2 × 10^8^ CFU) at 6 and 16 weeks of age. Group II chickens were primed and boosted with 200 μl of bacterial suspension containing 2 × 10^7^ CFU of SG 9R at 6 and 16 weeks of age. Group III chickens were inoculated with 200 μl of PBS at 6 and 16 weeks of age as the control group. For the bacteriological examination of eggs, eggs were collected daily for the four weeks (from 18 to 21 weeks of age) following the first egg laid by each chicken, and were examined for SG. Egg production of all hens was monitored for four weeks (from 22 to 25 weeks of age).

### Cell-mediated immune response

A lymphocyte proliferation assay (LPA) was performed in experiment 1 and 2 using specific antigens prepared from wild-type SG as previously described [8]. Peripheral blood samples were aseptically collected from five randomly selected birds per group, for three weeks after prime and boost immunization. Peripheral blood mononuclear cells (PBMCs) were separated using the Histopaque®-1077 (Sigma-Aldrich, St. Louis, MO, USA) according to the product information. Lymphocyte proliferation activity was measured using adenosine triphosphate (ATP) bioluminescence as a marker of cell viability using a ViaLight® Plus (Lonza Rockland, ME, USA). The blastogenic response of the assay was expressed as the mean stimulation index (SI), which was calculated by dividing the mean OD value of the culture stimulated with the antigen by the mean OD value of the non-stimulated culture [11].

### Humoral immune responses

Indirect enzyme-linked immunosorbent assay (ELISA) was performed using an outer membrane protein (OMP) fraction extracted from the wild-type SG strain, JOL420 [8]. Plasma samples were separated by centrifugation of wing vein peripheral blood to determine plasma immunoglobulin G (IgG) concentrations. Intestinal lavage samples were collected using pilocarpine-based lavage method [12]. All plasma and intestinal lavage samples were collected biweekly in experiment 1. In addition, all plasma and intestinal lavage samples were collected at 0, 3, 8 wppi and 3 wpbi in experiment 2. Evaluation of plasma IgG and intestinal secretory immunoglobulin A (sIgA) concentrations was performed as previously described [12] using chicken IgG and IgA ELISA Quantitation Kits (Bethyl Laboratories, TX, USA) according to the product information. Microlon® ELISA plate wells (Greiner Bio-One GmbH, Frickenhausen, Germany) were coated with 100 μl of OMP at a concentration of 0.2 mg/ml. Wells were reacted with plasma and intestinal lavage samples at dilutions of 1:250 and 1:100, respectively, for 1 hr, followed by reaction with HRP-conjugated goat anti-chicken IgG and IgA at dilutions of 1:100,000 and 1:60,000, respectively, for 1 hr.

### Protection efficacy

Chickens in all experiment 1 groups were orally challenged with 200 μl of bacterial suspension containing 1 × 10^6^ CFU of the wild-type SG strain JOL420, at 4 weeks post-boost-immunization (wpbi). Mortality was assessed daily for 14 days post challenge. All remaining animals were euthanized on 14 days post-challenge (dpc) for postmortem examination. Gross lesions in the livers and spleens were observed and scored as previously described [13].

### Observation of general condition and body weight after immunization

In experiment 2, general body condition, clinical symptoms, and mortality were observed daily from 6 to 25 weeks of age, for 19 weeks after prime-immunization. In addition, the body weights of all chickens were recorded at 5, 9, 13, 17, and 21 weeks of age.

### Egg production and bacteriological examination of eggs after immunization

Eggs were collected daily, and the egg production rate was measured to assess the influence of immunization on egg production daily from 22 to 25 weeks of age in experiment 2. Weekly egg production rates were calculated as follows: egg production rate = (the total number of eggs laid)/(the number of hens × 7 days). The bacteriological examination of eggs was performed from 18 to 21 weeks of age as previously described, with slight modification [14,15]. Briefly, eggs of all chickens in each group were pooled in one batch so that the number of eggs per batch varied between one and six. Upon collection, feces on the surfaces of the eggs were removed, and the eggs were decontaminated by dipping in 95% ethanol for 1 min. The eggs were broken aseptically, and the total content of the eggs was pooled and homogenized with sterilized wooden sticks. A volume of 40 ml of buffered peptone water (BPW) (Becton, Dickinson and Company, Sparks, MD, USA) per egg was added to the pooled egg content and incubated at 37°C for 24 hr. Subsequently, 300 μl of the culture was transferred to 3 ml Rappaport-Vassiliadis R10 (RV) broth (Becton, Dickinson and Company, Sparks, MD, USA) and incubated at 40°C for 48 hr. A loop of the enriched broth was streaked onto Brilliant Green Agar (BGA) (Becton, Dickinson and Company, Sparks, MD, USA), and *Salmonella*-type colonies were examined after overnight incubation at 37°C. Positive colonies were confirmed with PCR using OMPC primers for the *Salmonella* genus, SG primers for *S*. Gallinarum, *lon* and *cpxR* primers for the vaccine strain, and LTB primers for the LTB adjuvant strain (Table 2) [8,16-18].

**Table 2:** Primers used in this study

**Primer**	**Primer sequence (5’- 3’)**	**Source**
OMPC-F	ATCGCTGACTTATGCAATCG	Alvarez et al. (2004)
OMPC-R	CGGGTTGCGTTATAGGTCTG	
SG-F	GATCTGCTGCCAGCTCAA	Kang et al. (2011)
SG-R	GCGCCCTTTTCAAAACATA	
lon-both-F	ATTTTATCTCCCCTTTCGTTTTTC	Jeon et al. (2012)
lon-both-R	CTGCCAGCCCTGTTTTTATTAGC	
cpxR-both-F	CAGCGCCAGCGTCAACCAGAAGAT	Jeon et al. (2012)
cpxR-both-R	GAGGCCATAACAGCAGCGGTAACT	
LTB-F	GCTCCCCAGTCTATTACAG	Hur and Lee (2011)
LTB-R	CTAGTTTTCCATACTGATTG	

### Statistical analysis

Statistical analyses were performed with SPSS 16.0 (SPSS Inc., Chicago, IL, USA). All results are expressed as means ± standard error of the mean (SEM), unless otherwise specified. Comparisons between the immunized and control groups were made using the Mann–Whitney *U* test. The chi-square test was performed for significant differences of mortality post-challenge between the immunized and control groups. Statistical significance was identified as *p*-value < 0.05.

## Results

### General condition and immune responses

**Figure 1:**
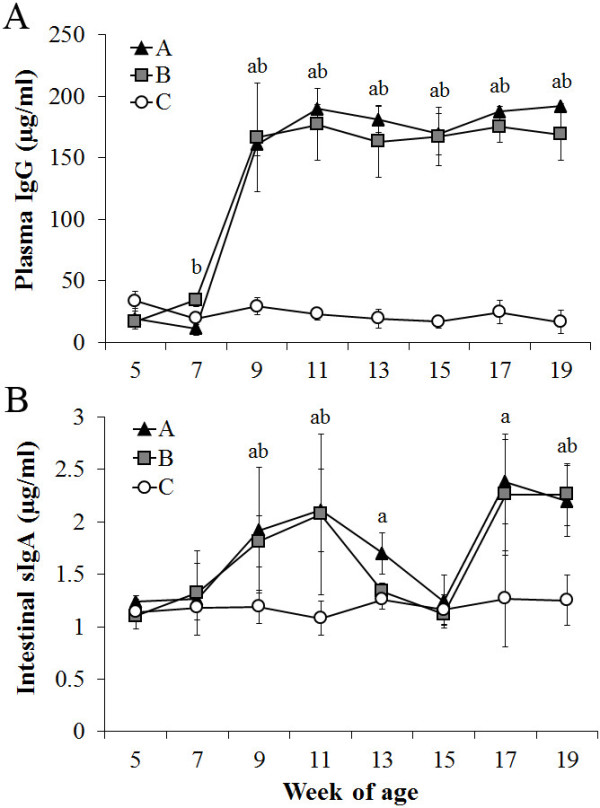
**Antibody responses to the SG mutant JOL916 and the LTB adjuvant strain JOL1229.** Antibody responses to SG specific antigen in chickens, orally prime-boost immunized with the vaccine candidate strain (JOL916) and/or the LTB adjuvant strain (JOL1229). Group A chickens were primed and boosted with a mixture consisting of four parts JOL916 (0.8 × 10^8^ CFU) and one part JOL1229 (0.2 × 10^8^ CFU), group B chickens were primed with a mixture consisting of four parts JOL916 (0.8 × 10^8^ CFU) and one part JOL1229 (0.2 × 10^8^ CFU), and were boosted with JOL916, and group C chickens were inoculated with PBS as the control group. (**A**) Plasma IgG concentration (μg/ml); (**B**) Secretory IgA concentration (μg/ml) in intestinal lavage. Lower case letters indicate a significant difference (*p* < 0.05) between each immunized and control group (a: Group A, b: Group B).

**Figure 2:**
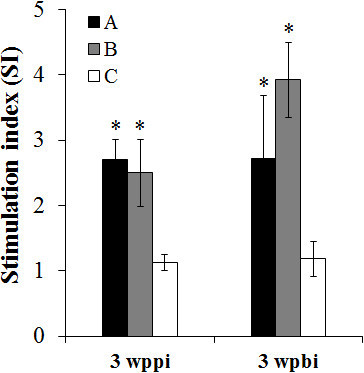
**Antigen-specific T lymphocyte proliferation to *Salmonella *Gallinarum.** Antigen-specific proliferation of lymphocytes from peripheral blood in chickens, orally prime-boost immunized with the vaccine candidate strain (JOL916) and/or the LTB adjuvant strain (JOL1229) using soluble antigen from the wild-type SG. Groups A to C are referred to in Figure 1. Asterisks indicate a significant difference between each immunized and control group (*p* < 0.05). wppi = week post-prime-immunization; wpbi = week post-boost-immunization.

No obvious clinical signs were found after oral prime and boost immunization at 6 and 16 weeks of age, respectively. There was no evidence of mortality in any of the chickens after inoculation with the JOL916-JOL1229 mixture or with JOL916 via oral route. Plasma IgG and intestinal sIgA concentrations were determined biweekly with indirect ELISA using specific SG antigen. Antibody responses to the *Salmonella* antigen in the plasma and intestinal lavage samples of the immunized and control chickens are shown in Figure 1. Plasma IgG concentrations in the chickens from all immunized groups were significantly elevated compared to the PBS control group after prime- and boost-immunization (*p* < 0.05) (Figure 1A). Intestinal sIgA concentrations in the chickens from group A were significantly elevated to 1.6-, 2.0-, and 1.4-fold compared to the control chickens at 3, 5, and 7 weeks post-prime-immunization (wppi), respectively (*p* < 0.05). In group B, intestinal sIgA concentrations were significantly elevated 1.5- and 1.9-fold compared to the control chickens at 3 and 5 wppi, respectively (*p* < 0.05). After boost immunization, the intestinal sIgA values of the chickens in group A were 1.9- and 1.8-fold higher compared to the corresponding control group, and the mean sIgA value of the chickens in group B was 1.8-fold higher than that of the control group (*p* < 0.05) (Figure 1B). Cell-mediated immune responses were examined using a lymphocyte proliferation response assay (LPA) with specific antigen extracted from wild-type SG at 3 wppi and 3 wpbi. Lymphocyte proliferation responses to the SG antigen revealed a significant increase in the stimulation indices (SIs) of all of the immunized groups compared to the control group (*p* < 0.05). In addition, the SI value of group B was approximately 1.4-fold higher than that of Group A at 3 wpbi (Figure 2).

### Protection efficacy against virulent challenge

Chickens from each group were orally challenged with 1 × 10^6^ CFU of virulent wild-type SG JOL420 at 4 wpbi. Chickens in all immunized groups were markedly protected against FT (*p* < 0.05) (Table 1). The mortality rates in Groups A, B, and C were 10%, 10%, and 70%, respectively. On 14 dpc, all surviving chickens were sacrificed for postmortem examination. The control group demonstrated severe pathological gross lesion scores of 2.1 ± 1.4, 2.1 ± 1.4, 2.1 ±1.4 and 2.3 ±1.3 for liver enlargement, liver necrotic foci, spleen enlargement and spleen necrotic foci, respectively (Table 1). However, lesion scores in all of the immunized groups were significantly lower than those of the control group (*p* < 0.05 or *p* < 0.01) (Table 1).

### General condition and body weight increase after immunization

**Figure 3:**
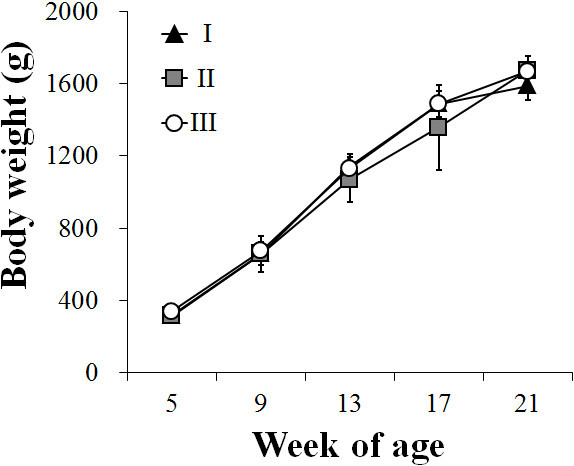
**Body weight increase in chickens immunized with the JOL916-JOL1229 mixture and SG 9R.** Body weight increase in group I to III; group I chickens were orally primed and boosted with 1 × 10^8^ CFU of the mixture consisting of four part JOL916 (0.8 × 10^8^ CFU) and one part JOL1229 (0.2 × 10^8^ CFU) at 6 and 16 weeks of age, group II chickens were subcutaneously primed and boosted with 2 × 10^7^ CFU of SG 9R at 6 and 16 weeks of age, group III chickens were inoculated with PBS at 6 and 16 weeks of age as the control group.

Oral immunization with the JOL916-JOL1229 mixture or with SG 9R in the chickens showed no clinical signs such as anorexia, depression, or diarrhea till the end of the experiment. The weight gains of both the immunized and negative control groups from 5 to 21 weeks of age are shown in Figure 3. No significant differences were observed among the groups, and the mean increases at 21 weeks of age were 1587.4 ± 80.3 g, 1673.6 ± 80.4 g, and 1667.6 ± 38.3 g in groups I, II, and III, respectively.

### Immune responses induced by the JOL916-JOL1229 mixture compared with those induced by a commercial vaccine

The cell-mediated immune responses induced by our mixture vaccine and SG 9R were examined using LPA on 3 wppi and 3 wpbi. All tested chickens in the immunized groups showed significantly elevated SI values compared to those in the control group (*p* < 0.05); SI values on 3 wppi were 2.6 ± 1.0, 2.1 ± 0.2, and 1.2 ± 0.4 in groups I, II, and III, respectively, and SI values on 3 wpbi were 2.4 ± 0.7, 2.3 ± 0.3, and 1.1 ± 0.1 in groups I, II, and III, respectively (Table 3). From 3 wppi to 3 wpbi, plasma IgG concentrations were significantly elevated in all immunized groups (groups I and II) compared to the control group (*p* < 0.01) (Table 3). No significant differences in SI values or plasma IgG levels were observed between our mixture group and the SG 9R group during the experiment. However, intestinal sIgA titers in chickens from group I were 1.9 times higher than those in control chickens on both 3 wppi and 3 wpbi (*p* < 0.05), while the mean increases in intestinal IgA levels in group II were not statistically significant compared to the control group (Table 3).

**Table 3:** SG-antigen-specific immune responses after prime -boost immunization in experiment 2

**Group**	***n***	**LPA**		**Plasma IgG (μg/ml)**				**Intestinal sIgA (μg/ml)**			
		**3 wppi**	**3 wpbi**	**0 wppi**	**3 wppi**	**8 wppi**	**3 wpbi**	**0 wppi**	**3 wppi**	**8 wppi**	**3 wpbi**
I	5	2.6±1.0^*^	2.4±0.7^*^	73.8±32.9	182.9±9.9^**^	138.5±12.2^**^	174.6±29.5^**^	4.3±0.6	9.3±0.8^*^	5.5±0.7	8.9±1.3^*^
II	5	2.1±0.2^*^	2.3±0.3^*^	75.7±43.9	171.7±23.2^**^	132.2±5.2^**^	177.8±14.0^**^	4.0±0.2	5.5±0.3	5.7±0.3	6.5±0.6
III	5	1.2±0.4	1.1±0.1	41.4±11.5	40.1±18.3	30.5±12.1	36.5±17.7	4.5±0.6	4.8±0.2	4.3±0.1	4.6±0.4

Group I chickens were primed and boosted with a JOL916-JOL1229 mixture, group II chickens were primed and boosted with SG9R, and group III chickens were inoculated with PBS as a control. LPA, lymphocyte proliferation assay. wppi, week post-prime-immunization. wpbi, week post-boost-immunization. Values represent stimulation index mean ± SEM. Asterisks indicate a significant difference compared to the control group (^*^p < 0.05, ^**^p < 0.01). *n*, numbers of the chickens used.

### Egg production after immunization

In experiment 2, the egg production rate of the immunized groups was observed from 22 to 25 weeks of age. The average overall egg production rate was 91.7% for chickens immunized with our strains, 92.5% for those immunized with SG 9R, and 93.1% for unimmunized chickens; there were no significant differences in the number of eggs laid by immunized hens (Table 4).

**Table 4:** Egg production rate in the immunized and unimmunized groups in experiment 2

**Group**	**Week of age**				**Averages**
	**22**	**23**	**24**	**25**	
I	90.0^a^	93.7	91.7	91.7	91.7
II	91.4	92.9	90.5	95.3	92.5
III	90.0	95.7	94.3	92.5	93.1

Group I chickens were primed and boosted with a JOL916-JOL1229 mixture, group II chickens were primed and boosted with SG9R, and group III chickens were inoculated with PBS as a control.

^a^Values represent the percentages of egg production calculated as described in the section of methods.

### Isolation of the vaccine strain from egg contents after immunization

In experiment 2, eggs from the immunized chickens with our vaccine strains were collected daily from 18 to 21 weeks of age, and examined for detection of *Salmonella*. Over the four weeks of the experiment, our vaccine strains were not detected in any eggs from the immunized chickens.

## Discussion

The prime-boost immunization strategy can elicit long-lasting humoral, mucosal, and cellular responses against a variety of antigens [19]. In the present study, we evaluated protection efficacy and immune responses in layer chickens, which had been immunized with a double immunization in order to optimize the efficacy of the vaccine. In the first experiment, group A chickens were primed and boosted with a mixture consisting of JOL916 and JOL1229, and group B chickens were primed with the same mixture and boosted with JOL916 alone. Both immunized groups showed a significant increase in the intestinal sIgA levels after prime and boost immunization, and this result indicates that the oral immunizations can stimulate antigen-specific mucosal immunity (*p* < 0.05) (Figure 1B). The mucosal immune defense is largely mediated by sIgA antibodies, which are the first line of defense against microorganisms and operate through immune exclusion [20,21]. Mucosal immunizations such as oral inoculations can be an effective means of inducing sIgA, as well as systemic antibodies and cell-mediated immune responses [22]. Plasma IgG antibody levels in the immunized groups were also significantly increased compared to those in the control group (*p* < 0.05) (Figure 1A). Systemic antibodies clear *Salmonella* from the blood, and also promote phagocytosis of *Salmonella* by opsonization method [23,24]. Significant cell-mediated immune responses were shown by LPA in both immunized groups compared to the control group at 3 wppi and 3 wpbi (*p* < 0.05) (Figure 2). It is widely accepted that enhancement of cellular immunity is crucial for protection against a primary *Salmonella* infection [25]. In addition, Th1-dominated cell-mediated responses are likely to be more important in the clearance of SG, which is believed to survive and multiply within macrophages [3,26]. All immunological data indicate that oral immunization with the LTB strain with a prime-boost strategy can effectively enhance and extend considerable levels of acquired immunity, including the mucosal immune responses.

Generally, oral tolerance has been defined as the specific suppression of cell-mediated and/or humoral immune responses to antigens by prior oral administration of the antigen [27]. Oral administration of antigens with recombinant enterotoxin B subunits such as LTB and cholera toxin B subunit (CTB) has been found to induce tolerance to the same antigens when subsequently inoculated [28]. In the experiment 1, chickens in groups A and B were both orally inoculated with a JOL916-JOL1229 mixture at 6 weeks of age. Group A chickens were orally boosted with the same mixture, while group B chickens were orally boosted with JOL916 alone at 16 weeks of age. Prime and boost immunizations offered significant protection efficacy against a wild-type SG challenge (*p* < 0.05), while 70% mortality was observed in the unimmunized control group (Table 1). In addition, the gross lesion scores of internal organs in the immunized groups were significantly lower than those of the control group (*p* < 0.05) (Table 1). Very mild or no gross lesions in the liver and spleen in the immunized groups may indicate that the acquired immunity successfully controlled FT infection after the booster inoculation. In addition, there were no statistically significant differences in the protection efficacy or immune responses between groups A and B (Figures 1 and 2, and Table 2), which suggests that oral tolerance may not be induced by boost inoculation with the LTB adjuvant strain. It is possible that the time interval (10 weeks) between the prime and boost inoculations can be a factor in the evasion of an oral tolerance event.

In the experiment 2, we compared the immunogenicity, body weight, and egg production rates of the chickens immunized with the JOL916-JOL1229 mixture to those immunized with SG 9R, a commercial SG vaccine. The cell-mediated immune responses and the systemic IgG responses were significantly increased in both immunized groups compared to the control group (*p* < 0.05 or *p* < 0.01) (Table 3). However, the sIgA concentrations of the chickens immunized with SG 9R were not significantly elevated compared to the control group (Table 3), while a significant elevation in intestinal sIgA in chickens immunized with our mixture was observed at 3 wppi and 3 wpbi (*p* < 0.05). The body weights of all immunized chickens were monitored for 16 weeks (5 to 21 weeks of age) after vaccination. There were no significant differences in body weight gain among the groups (Figure 3). The egg production rate after vaccination was also examined in the immunized groups and control group for four weeks (22 to 25 weeks of age). There was no statistical difference in number of eggs laid by the double immunized chickens with our vaccine mixture, with SG 9R strain, and the unimmunized chickens (Table 4). These data suggest that both immunized groups showed similar results in the body weight gain, egg production rates and systemic IgG immune response. However, immunization with only JOL916-JOL1229 mixture showed enhanced intestinal sIgA immune response, which may be important to mediate antibody-dependent T-cell-mediated cytotoxicity against *Salmonella*[29].

In FT infection, chickens may infect their own, as well as succeeding generations through egg transmission [2]. In the present study, bacteriological examination of the total content of the eggs was performed to determine the safety of the JOL916-JOL1229 mixture. The SG mutant and/or LTB adjuvant strains were not detected in any egg of the immunized chickens during four weeks (18 to 21 weeks of age), which suggests that immunization with the JOL916-JOL1229 mixture does not cause bacterial contamination of the ovum.

## Conclusion

In conclusion, data from both the experiments indicate that the immunizations with the JOL916-JOL1229 can be safe; and can induce acquired immunity including enhanced mucosal immunity. Furthermore, double immunization with the JOL916-JOL1229 may trigger elevated levels of immune responses to optimize the protection efficacy against FT infection in hens.

## Abbreviations

ATP: Adenosine triphosphate; BGA: Brilliant Green Agar; BPW: Buffered peptone water; CFU: Colony-forming units; CTB: Cholera toxin B subunit; Dpc: Day post-challenge; ELISA: Enzyme-linked immunosorbent assay; FT: Fowl typhoid; GM1: Monosialotetrahexosylganglioside; HRP: Horseradish peroxidase; IgG: Immunoglobulin G; LB: Luria-Bertani; LPA: lymphocyte proliferation assay; LTA: *Escherichia coli* heat-labile enterotoxin A subunit; LTB: *Escherichia coli* heat-labile enterotoxin B subunit; OD: Optical density; OMP: Outer membrane protein; PBMCs: Peripheral blood mononuclear cells; PBS: Phosphate-buffered saline; RV: Rappaport-Vassiliadis R10; SEM: Standard error of the mean; SG: *Salmonella enterica* serovar Gallinarum biovar Gallinarum; SG 9R: Attenuated rough strains of *Salmonella* Gallinarum strain 9; SI: Stimulation index; sIgA: Secretory Immunoglobulin A; wpbi: Week post-boost-immunization; wppi: Week post-prime-immunization.

## Competing interests

The authors declare that they have no competing interests.

## Authors’ contributions

BWJ and JHL designed the experiment and co-wrote the manuscript. BWJ performed all the experiments. RMN assisted with immunological experiments and protection efficacy experiment. All authors read and approved the manuscript.
